# Human neural stem cell transplantation improves cognition in a murine model of Alzheimer’s disease

**DOI:** 10.1038/s41598-018-33017-6

**Published:** 2018-10-03

**Authors:** Lisa M. McGinley, Osama N. Kashlan, Elizabeth S. Bruno, Kevin S. Chen, John M. Hayes, Samy R. Kashlan, Julia Raykin, Karl Johe, Geoffrey G. Murphy, Eva L. Feldman

**Affiliations:** 1 Department of Neurology, University of Michigan, Ann Arbor, MI USA; 2 Department of Neurosurgery, University of Michigan, Ann Arbor, MI USA; 3 Department of Biomedical Engineering, Georgia Institute of Technology, Atlanta, GA USA; 4 Neuralstem, Inc, Germantown, MD USA; 5 Department of Molecular & Integrative Physiology, Molecular & Behavioral Neuroscience Institute, University of Michigan, Ann Arbor, MI USA

## Abstract

Stem cell transplantation offers a potentially transformative approach to treating neurodegenerative disorders. The safety of cellular therapies is established in multiple clinical trials, including our own in amyotrophic lateral sclerosis. To initiate similar trials in Alzheimer’s disease, efficacious cell lines must be identified. Here, we completed a preclinical proof-of-concept study in the APP/PS1 murine model of Alzheimer’s disease. Human neural stem cell transplantation targeted to the fimbria fornix significantly improved cognition in two hippocampal-dependent memory tasks at 4 and 16 weeks post-transplantation. While levels of synapse-related proteins and cholinergic neurons were unaffected, amyloid plaque load was significantly reduced in stem cell transplanted mice and associated with increased recruitment of activated microglia. *In vitro*, these same neural stem cells induced microglial activation and amyloid phagocytosis, suggesting an immunomodulatory capacity. Although long-term transplantation resulted in significant functional and pathological improvements in APP/PS1 mice, stem cells were not identified by immunohistochemistry or PCR at the study endpoint. These data suggest integration into native tissue or the idea that transient engraftment may be adequate for therapeutic efficacy, reducing the need for continued immunosuppression. Overall, our results support further preclinical development of human neural stem cells as a safe and effective therapy for Alzheimer’s disease.

## Introduction

Alzheimer’s disease (AD) is the most prevalent age-related neurodegenerative disorder and leading cause of dementia, affecting over five million people in the U.S.^1^. With increasingly aging populations, AD has become a significant public health concern^2^. Unfortunately, there is no cure or preventative measures, and although currently prescribed drugs temporarily slow dementia symptoms, they ultimately fail to alter the disease course. Consequently, huge efforts are underway to identify new treatments and multitudes of prospective new compounds have moved through the AD clinical development pipeline over the past decade^2–4^. However, the multifactorial and complicated pathophysiology of AD makes therapeutic advances very difficult. Most candidates fail to show efficacy compared to placebo groups in Phase 3 studies, and no new treatments have been approved for market since 2003^4^.

More recently, rapid advances in stem cell biology have led to an explosion of potential novel therapies for several neurodegenerative diseases. Stem cell-based therapies offer a particularly attractive alternative to single-target small molecules by providing a multifaceted approach to treat disease. Besides direct tissue replacement, stem cells can form synapses, modulate inflammation, and provide sustained enrichment of neuronal microenvironments via paracrine factor delivery. Direct central nervous system (CNS) stem cell injections are in clinical trials for stroke and Parkinson’s disease^5–7^, and our own laboratory has unique experience translating a cellular therapy to the clinic for amyotrophic lateral sclerosis (ALS)^8–13^. In AD, several preclinical studies have shown the short-term benefit of stem cell transplantation in murine models and indicate that efficacy is frequently associated with the delivery of neurotrophic factors^14–20^. This has supported a handful of ongoing clinical trials in AD patients assessing various mesenchymal stem cell types by intravenous and intraventricular delivery^21^ and one completed open-label Phase I trial showing safe intracranial targeting of the hippocampus^22^. Although these approaches are certainly promising for AD therapeutics, further study is needed to identify ideal stem cell candidates and define dosing and delivery parameters for optimal efficacy in patients.

With Neuralstem, Inc., we developed a unique line of human cortex-derived neural stem cells (NSCs; NSI-HK532-IGF-1), safely engineered to produce multiple neurotrophic factors^23^. We previously reported that this specific NSC line is neuroprotective *in vitro* and survives and migrates after *in vivo* grafting^23,24^. Furthermore, we have obtained investigational new drug status for the sister spinal cord-derived cell line of this NSC candidate (NSI-566RSC), currently in an FDA-approved clinical trial in ALS patients^13,25^. As a first step in preclinical development, we hypothesized that NSCs can influence multiple underlying pathologies associated with AD and improve cognitive deficits. Here, we describe the results of our first proof-of-concept efficacy study examining intracranial transplantation of this human NSC line in the APP/PS1 murine model of AD. To date, the majority of studies in AD mouse models have tested efficacy in the shorter-term, ranging from 1 to 6 weeks post-transplant^14,18,26^. We therefore performed longer-term efficacy testing, and assessed the impact of NSCs on cognition and disease pathology throughout a 17-week post-transplant period.

## Results

### NSCs targeted to the fimbria fornix improve cognitive function

**Figure 1:**
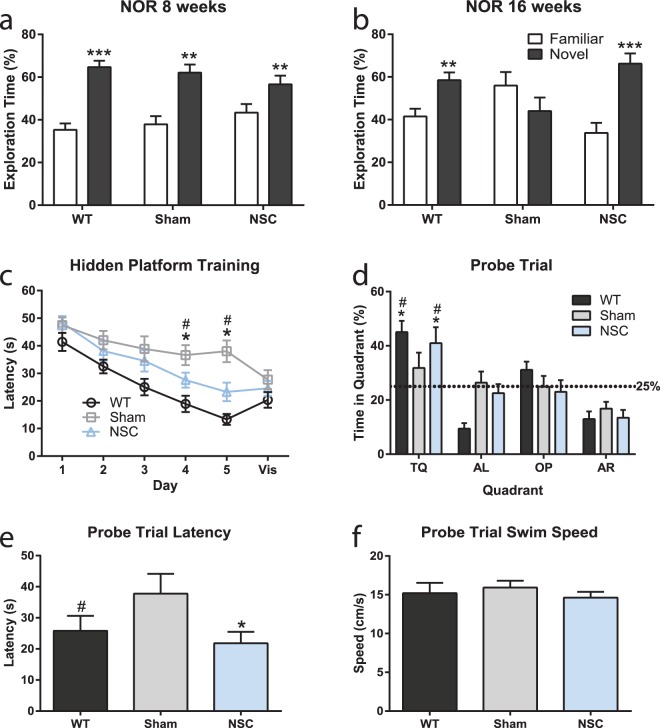
NSCs targeted to the fimbria fornix improve hippocampal-dependent cognition in APP/PS1 mice. Percent time exploring familiar and novel objects in the NOR test, preoperatively at 8 weeks (**a**) and postoperatively at 16 weeks (**b**). All groups performed the task normally at 8 weeks (**a**); however, at 16 weeks (4 weeks post-NSC/vehicle transplant), the sham group was unable to perform the task, whereas WT and NSC groups recognized the novel object and spent significantly more time exploring it (**b**; **p < 0.005 WT, ***p < 0.0001 NSC, novel vs. familiar; t-test). MWM at 28 weeks (16 weeks post-NSC/vehicle transplant); escape latency during 5 d hidden platform training (**c**; 6 trials per day) and 24 h probe trial quadrant statistics (**d**), escape latency (**e**) and swim speed (**f**). At days 4 and 5 of training, sham controls exhibited a deficit in the latency to find the hidden platform compared to WT (^#^p < 0.05; repeated measures ANOVA), while at these same time points, NSC mice exhibited improved performance compared to sham mice (*p < 0.05; repeated measures ANOVA) (**c**). In a 24 h probe trial, WT and NSC-treated mice exhibited a significant preference for the TQ over the other quadrants (AL, AR, and OP) (**d**; *p < 0.05; ANOVA) and over chance (**d**; ^#^p < 0.05; vs. chance; t-test with a hypothetical mean of 25%, indicated by dashed line), and reached the former platform location almost twice as fast as sham controls (**e**; *p < 0.05 NSC vs. sham; ^#^p < 0.05 WT vs. sham; ANOVA), demonstrating a strong memory for the former platform location. These data indicate that NSCs improved short-term non-associative memory as well as spatial reference and working memory. Data are mean ± SEM (*^#^p < 0.05, **p < 0.005, ***p < 0.0001). Sample size: WT n = 5 (NOR) and n = 14 (MWM in 2 cohorts), NSC n = 10, and sham n = 10.

To determine the impact on hippocampal-dependent short-term non-associative memory, we performed novel object recognition (NOR) testing before and after transplantation of 180k NSCs targeted to the fimbria fornix. Preoperatively at 8 weeks of age (Fig. 1a), all mice performed recognition testing normally, and as expected spent significantly more time exploring the novel vs. familiar object (p = 0.0001 wild-type (WT); p = 0.0008 sham; p = 0.003 NSC; novel vs. familiar). However, at 16 weeks of age, 4 weeks after NSC/vehicle transplant (Fig. 1b), we found that the vehicle-injected (sham) group was unable to distinguish the novel object (p = 0.1937), whereas WT controls and NSC-treated mice spent significantly more time exploring the novel object (p = 0.0042 WT; p = 0.0001 NSC; novel vs. familiar), demonstrating that NSC transplantation improved short-term non-associative memory.

In the Morris water maze (MWM) 16 weeks postoperatively (28 weeks of age), we compared spatial learning and long-term (24 h) memory between treatment groups. Here, we found significant differences between NSC-treated and sham controls in all parameters tested. Across a 5-day training period, sham-treated mice exhibited a deficit in the latency to find the hidden platform compared with the WT group (day 4, p = 0.0002; day 5, p = 0.0001; WT vs. sham). At the same time points, NSC-treated mice exhibited improved latencies compared to the sham group (Fig. 1c), indicating improvements in spatial acquisition (day 4, p = 0.0464; day 5, p = 0.0060; NSC vs. sham). NSC mice also demonstrated significantly improved performance compared to the sham group during a 24 h probe trial (Fig. 1d). Here, NSC mice showed a significant preference for the target quadrant (TQ) over the other quadrants (alternate left, AL; opposite, OP; alternate right, AR), and over chance (p = 0.0212, p = 0.0260, p = 0.0003 TQ vs. AL, OP, AR; p = 0.0249 TQ vs. 25%). NSC mice also exhibited decreased escape latency in the probe trial compared to the sham group (p = 0.0434; NSC vs. sham; Fig. 1e). These data demonstrate a strong memory for the platform’s previous location and improved spatial memory recall in the NSC mice. Swim speed and visible platform trials were unchanged between groups (Fig. 1c,f), suggesting that any observed deficits were not due to deficits in visual acuity, motivation, or motor performance.

### Transient NSC engraftment reduces amyloid beta (Aβ) plaque pathology

Survival and integration of transplanted NSCs was assessed in brain tissue harvested 17 weeks post-transplant. No human NSCs were detected in NSC mice by immunohistochemistry (IHC) for human specific markers (data not shown).

**Figure 2:**
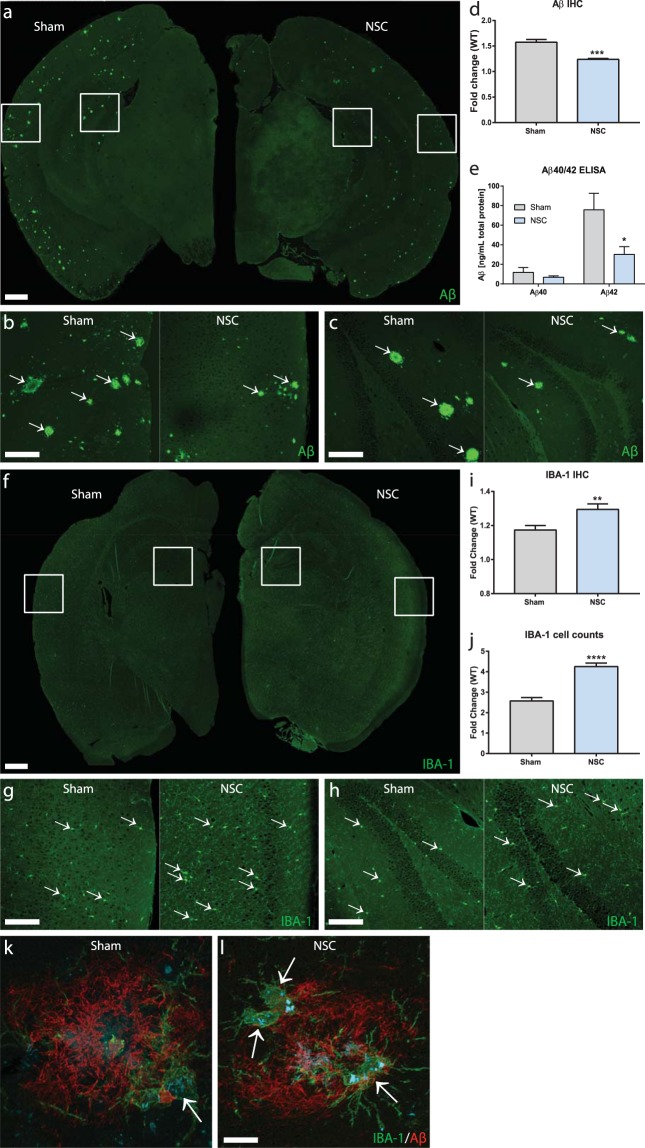
NSC transplantation is associated with reduced Aβ load and increased microglia within hippocampal and cortical regions. Representative IHC images of Aβ (**a**, scale bar 500 µm; **b**,**c**, scale bar 200 µm) and IBA-1 (**f**, scale bar 500 µm; **g**,**h**, scale bar 200 µm) in hippocampus and cortex of APP/PS1 mice at 29 weeks (17 weeks post-NSC/vehicle transplant). Significant plaque formation was evident in the sham group, and was reduced in NSC-treated mice (arrows, **a**–**c**). Quantification by IHC fluorescence intensity (**d**) and by Aβ40/42 ELISA on whole brain homogenate (**e**) shows a significant reduction in Aβ level in the NSC group compared to sham controls (p < 0.05; Mann-Whitney, t-test). The NSC group also exhibits many IBA-1+ microglia with activated morphologies (arrows, fold change relative to WT controls; **f**–**h**), which was increased compared to sham controls (p < 0.05; t-test) when quantified by fluorescence intensity (**i**) and number of activated cells (**j**). In NSC mice, many microglia (green, arrows) localized to and were intimately associated with Aβ plaques (red) compared to the sham group (**k**,**l**; scale bar 50 µm). Data are representative images or mean ± SEM (*p < 0.05). Sample size: WT n = 5, NSC n = 10, and sham n = 10.

Since cognitive function was improved in NSC-treated mice despite the absence of engrafted transplanted cells, we next determined the impact of NSC transplantation on Aβ pathology by quantitating Aβ plaque load in animals that completed behavioral testing. In quantification of Aβ by IHC (Fig. 2a–d), we detected significantly decreased plaque load in both hippocampal and cortical brain regions in the NSC group compared to sham controls (p = 0.0001; NSC vs. sham). Enzyme-linked immunosorbent assay (ELISA) analysis of whole brain homogenate (Fig. 2e) showed a similar decrease of approximately 50% in total Aβ42 in whole brains (p = 0.0136; NSC vs. sham). Aβ40 levels were unchanged (p = 0.1971; Fig. 2e).

We also examined if the observed improvements in cognition could be associated with changes in synaptic density or the cholinergic system. In IHC quantification, we observed no differences between groups in pre-synaptic synaptophysin (p = 0.1297; NSC vs. sham), post-synaptic postsynaptic density protein 95 (PSD-95; p = 0.4580; NSC vs. sham), or choline acetyltransferase (ChAT; p = 0.1539; NSC vs. sham) (Supplementary Fig. S1a–d).

### NSCs modulate microglial activation *in vivo* and *in vitro*

Inflammatory processes, including the microglial response, are closely related to amyloidosis and AD pathogenesis. To determine whether NSCs had an immunomodulatory effect on microglia, we analyzed IBA-1+ (ionized calcium binding adaptor molecule-1) cell number and total IBA-1 levels by IHC (Fig. 2f–j). We found total IBA-1 expression levels were increased in the NSC group compared to sham controls (p = 0.0098; NSC vs. sham), as well as numbers of IBA-1+ microglia exhibiting an activated morphology, characterized by a large ameboid cell body and thicker processes (p = 0.0001; NSC vs. sham). Aβ/IBA-1 co-staining revealed infiltrating microglia at the plaque site, with increased recruitment of microglia to Aβ plaques in the NSC group (Fig. 2k). These data suggest that NSCs promote a neuroprotective phenotype of microglia that may assist in clearing toxic amyloid aggregates. We next measured expression levels of pro-inflammatory cytokines interleukin 1 beta (IL-1β) and tumor necrosis factor alpha (TNF-α) by ELISA (Supplementary Fig. S1e–f), but levels were unchanged in the NSC group (IL-1β; p = 0.4082 and TNF-α; p = 0.2115; NSC vs. sham), suggesting that NSCs modulated microglia function independent of these pro-inflammatory mediators.

**Figure 3:**
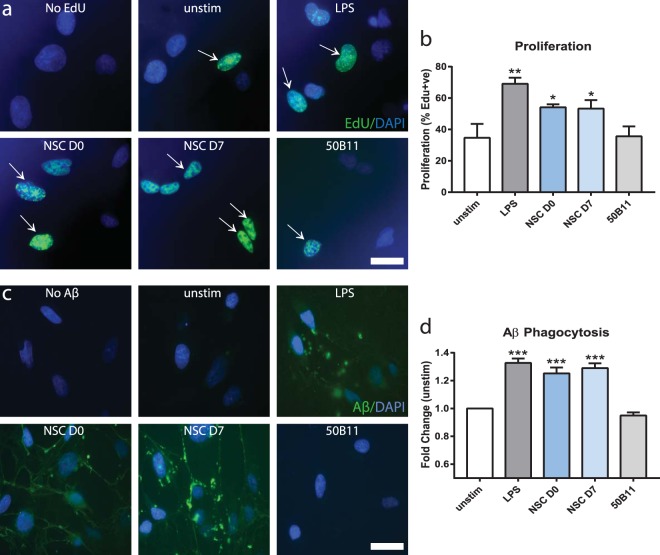
NSCs modulate microglial function *in vitro*: a possible therapeutic mechanism. Representative immunocytochemistry images and quantification of *in vitro* microglial proliferation (**a**,**b**) and phagocytosis of Aβ (**c**,**d**) with and without co-cultured NSCs. Proliferating EdU+ microglia (green) were increased by addition of LPS and co-culture with undifferentiated and differentiated NSCs vs. unstimulated and 50B11 controls (**a**). Quantified percentage proliferation was significantly increased in NSC co-cultures vs. the control groups (**b**; p < 0.05; ANOVA). NSCs also enhanced phagocytic activity of microglia (**c**), evidenced by increased accumulation of fluorescently-labeled Aβ vs. unstimulated and 50B11 controls. Quantified phagocytosed fluorescence was significantly increased by addition of LPS and NSC co-cultures vs. unstimulated and 50B11 controls (**d**; p < 0.0001; ANOVA). Data are representative images or mean ± SEM (*p < 0.05, ***p < 0.0001) of 3–5 independent experiments (n = 3–6 per condition). Scale bar 50 µm. Abbreviations: unstim, unstimulated; D0, day 0 undifferentiated NSCs; D7, day 7 differentiated NSCs; EdU, 5-ethynyl-2′-deoxyuridine.

To determine whether NSCs can directly modulate microglia function, microglial proliferation and phagocytosis of Aβ was assessed in a series of *in vitro* co-culture assays. Microglial proliferation (Fig. 3a,b) was significantly increased by approximately 20% in NSC co-cultures, compared to unstimulated resting microglia and a control cell type, 50B11 (p = 0.03; NSC vs. unstimulated, p = 0.049; NSC vs. 50B11). In an Aβ phagocytosis assay, NSCs also promoted phagocytic activity (Fig. 3c,d), evidenced by significantly increased accumulated fluorescent Aβ, compared to unstimulated and 50B11 controls (p = 0.0001; NSC vs. unstimulated and vs. 50B11). NSC effects on microglial proliferation and phagocytosis were at similar levels to lipopolysaccharide (LPS)-positive control groups and did not significantly differ in either assay. These data indicate that NSCs modulate microglial function *in vivo* and *in vitro*.

## Discussion

Thus far, conventional pharmacological approaches have failed to produce improvements in AD, indicating that single-target approaches are insufficient to address the complex disease process^2–4,27^. Given that stem cell therapies target multiple disease mechanisms, we examined the long-term efficacy of NSI-HK532-IGF-1, a human NSC line that we previously characterized *in vitro*^23^, in the APP/PS1 AD mouse model. In line with previous transplantation studies of this particular cell line^24^, this study supports the safety and feasibility of intracranial transplantation of human NSCs, with no adverse findings throughout the 17-week post-transplant period. Moreover, NSC transplantation targeted to the fimbria fornix significantly improved hippocampal-dependent cognitive function and was associated with reduced plaque load and increased microglial activation, but with no observable tissue integration or impact on synaptic density or cholinergic function.

Several studies report therapeutic benefits of cell therapies in murine AD models delivered by various routes of administration including intravenous^28–31^, intraventricular^17^ and intracranial^14,16,26,32^ injection. With intravenous delivery, engraftment can be hindered by the ability of transplanted cells to pass through the blood-brain barrier and reach the brain structures most affected in AD^28,31^. This method generally results in reduced efficacy and limited donor cell engraftment when compared to direct injection approaches^14,16,26,28–32^. Direct stereotactic injection permits targeting of specific intracranial structures within the brain and is proven safe through the routine image-guided techniques used in current human neurosurgical procedures, such as deep brain stimulation probe placement and more recently for stem cell delivery in stroke^5,6^. We are particularly interested in targeting the fimbria fornix, a large white matter tract connecting the hippocampus to cortical and subcortical brain structures, in order to provide therapeutic access to memory and learning centers of the brain affected early in AD, and facilitate efficacy assessment by hippocampal-based cognitive tests. In this first long-term efficacy study, our candidate NSCs enhanced recognition memory in NOR 4 weeks post-transplant and improved performance in training and probe phases of the MWM 16 weeks post-transplant. These data indicate that NSC targeting to the fimbria fornix of the hippocampus at a dose of 0.9 × 10^5^ cells per hippocampus, enhances short-term memory as well as spatial learning and long-term memory consolidation. Improved spatial memory has been shown by others in APP/PS1 mice 5 weeks after repeated intracerebral injections of human umbilical cord-derived mesenchymal stem cells (three bilateral injections at 2-week intervals; 3 × 10^5^ cells per hippocampus)^16^. Human fetal-derived stem cells also produced improvements in spatial learning and memory of NSE-APP mice 6 weeks after intraventricular injection (bilateral; 5 × 10^5^ cells per ventricle)^17^. More recently, Ager *et al*. demonstrated a similar effect for intrahippocampal delivery of human CNS stem cells in the 3XTg-AD model (bilateral; 1 × 10^5^ per hippocampus), although behavioral testing was evaluated only 4 weeks post-transplant^14^. And so, while consistent with other studies showing improved spatial memory in the MWM following intrahippocampal stem cell delivery^14,16,17^, to our knowledge, our study is the first to show robust longer-term effects on cognition (greater than 6 weeks) after transplantation of clinically applicable human NSCs in immunosuppressed AD mice.

As cognitive changes are often associated with altered Aβ pathology^16,19,33–35^, we examined Aβ levels after behavioral testing. In line with reports correlating Aβ reduction with functional and cognitive improvement^16,19,33,34^, in NSC mice we found decreased plaques and lower total Aβ42, the main component of neuritic plaques. Interestingly, there was no impact on synapse density or cholinergic neurons, as has been reported by other groups^14,15,17,36,37^. Rather, NSC transplantation was associated with altered activity of brain microglia in cortex and hippocampal regions. In the CNS, microglia are heterogeneous and diverse in their function, with phenotypes ranging from pro-inflammatory to immunosuppressive. Microglia secrete proteolytic enzymes that can degrade Aβ, such as neprilysin, insulin-degrading enzyme, and matrix metalloproteinases (MMPs), and express receptors that can facilitate Aβ phagocytosis and clearance^19,38–40^. In AD, microglia-mediated Aβ clearance pathways are dysregulated^41^. We therefore hypothesized that NSCs may indirectly assist in the clearance of toxic Aβ aggregates by promoting a more neuroprotective microglial phenotype in the brain. Our *in vitro* data confirmed that NSCs can directly modulate the functional properties of microglia, and this premise is further validated by a number of reports indicating that mesenchymal stem cell types accelerate microglial activation^18,26,29,42,43^. Supporting this concept, impaired microglial recruitment via genetic ablation of toll-like receptor 2 (TLR2) in APP/PS1 mice and C-C chemokine receptor type 2 (CCR2) in Tg2576 mice accelerated memory impairment and increased Aβ42^44,45^. Although some transplantation studies have reported pro-inflammatory mediators^17^, IL-1β and TNF-α expression levels were unchanged in NSC mice. The mechanism by which microglia become “alternatively activated” is more likely due to soluble factors produced by the stem cells themselves that enhance Aβ clearance pathways, such as MMPs, transforming growth factor beta (TGF-β), and chemokine ligands (CCLs)^43,46^. Identifying key factors and pathways is the focus of our current studies on the crosstalk between NSCs and host microglia.

Microglial activation through secreted NSC factors and subsequent Aβ clearance represents just one of many possible potential therapeutic mechanisms. Alternative explanations generally include NSC differentiation into glia or neuronal phenotypes and subsequent functional integration. In the present study, functional improvements and tissue changes occurred *in vivo*, although well-established PCR or IHC methods^14,23,24,47,48^ did not identify human stem cells post-mortem. Transplanted cell survival and engraftment is determined by multiple parameters including animal model, cell type, dose, route of administration and treatment duration. Since the majority of studies to date have assessed efficacy at much earlier time points, our experimental design investigated the longer-term impact of a well-characterized human NSC line and required an immunosuppression regimen. Using the same immunosuppression protocol and cell dose, we previously reported NSC survival 8 weeks post-transplant in APP/PS1 mice^24^, and in our preclinical ALS studies we have shown human spinal stem cell survival in similarly immunosuppressed rats^47,49^. In studies by others, human donor cell survival and integration is reported in various mouse models, although at much shorter time points ranging from 1 to 6 weeks post-transplant^14,17,30,32^. When the immunosuppression variable is eliminated through the use of immune privileged mesenchymal stem cell types, transplanted cells were undetectable in multiple studies, indicating that donor cell survival is not always robust^28,31^. Although intravenous approaches can limit engraftment within the brain, even with direct intrahippocampal injection, two studies were unable to identify donor human mesenchymal stem cells at 4 and 5 weeks post-transplantation in APP/PS1 mice (bilateral; 5 × 10^4^ cells per hippocampus^26^; three bilateral injections at 2-week intervals; 3 × 10^5^ cells per hippocampus^16^). Interestingly and in line with our results, improvements in cognition and AD pathology occurred in these studies although no donor cells were present at 4 and 5 week endpoints.

Recent reports more closely paralleling our study design detail the transplantation of human progenitor cell types requiring immunosuppression^14,17^. In 13 month old NSE-APP mice immunosuppressed with cyclosporine, high dose human NSCs (5 × 10^5^ cells per hippocampus) were detectable 6 weeks after intraventricular injection^17^. Ager *et al*. also identified engrafted human neural progenitor cells in 3xTg-AD and CaM/Tet-DT_A_ mice immunosuppressed with cyclosporine and an antibody cocktail targeting leukocyte costimulatory molecules, 4 weeks post-transplant at a comparable dose to ours (1 × 10^5^ cells per hippocampus)^14^. In the present study, at a much later time point of 16 weeks post-transplant, NSC-treated AD mice exhibited improved cognitive function, but no human-specific sequences or proteins were detected by PCR or IHC, despite adequate plasma levels of tacrolimus in all groups at the study endpoint (data not shown). This suggests that our immunosuppression protocol in combination with the particular mouse model does not support transplanted cell survival for longer than the 8-week time course we previously reported^23^. Additional study is needed to assess alternative immunosuppression protocols and identify ideal time courses for treatment.

However, the limited engraftment observed in our study is not wholly unexpected based on our reported and unreported findings after stem cell transplantation in man, where transplanted stem cells were undetectable with IHC after long survival times in one-third of ALS patients^12^. Taken together, these data are highly relevant for future clinical studies, where short-term survival of NSCs accompanied by a longer-term therapeutic benefit could possibly mitigate the need for continued immunosuppression^10^. Along these lines, the development of real-time non-invasive methods to track transplanted cells *in vivo* will significantly advance the development of stem cell therapies for AD. Tracking transplanted cells by serial MRI, for example, would enable immediate confirmation of graft delivery to intracranial targets as well as accurate monitoring and tracking throughout the study timeline. Clinically, it would also provide inclusion/exclusion criteria by identifying subjects with desired graft distributions, assisting with interpretation of trial failure or success. Additional preclinical studies utilizing such tracking technologies will allow us to track transplanted cells over extended time periods to identify the limits of cell survival *in vivo*, and assess alternative immunosuppression paradigms if necessary.

In conclusion, our findings show that transplantation of human NSCs targeted to the fimbria fornix is safe and improves behavioral and pathological phenotypes in the APP/PS1 model, partly via an immunomodulatory mechanism. Further long-term efficacy testing and real-time tracking in multiple preclinical models is required to comprehensively characterize grafted cells and identify key factors underlying therapeutic mechanisms. These data support additional preclinical study of stem cell candidates to develop an effective disease modifying intervention for AD patients.

## Materials and Methods

### Animals and NSC transplantation

All animal protocols were approved by the University of Michigan Institutional Animal Care and Use Committee (approval ID #PRO00006454), and performed in accordance with University of Michigan guidelines (accredited by the Association for the Assessment and Accreditation of Laboratory Animal Care International) and state and federal regulations. Mice were housed in groups of three to five, with a 14/10 h light/dark cycle, an ambient temperature of 20–22 °C, and *ad libitum* access to food and water. Male B6C3-Tg(APPswe/PSEN1ΔE9)85Dbo/J (APP/PS1; n = 20) mice (stock #034829-JAX; Jackson Laboratory, Bar Harbor, ME) were randomly assigned to experimental groups (sham n = 10; NSCs n = 10). Age-matched non-carrier male littermates B6C3F1/J served as WT controls (n = 14; in 2 cohorts) for behavior, ELISA, and IHC analyses.

Intracranial transplantation of NSCs was performed using the same approach described in our previous studies^23,24^. Briefly, at 12 weeks of age, mice were anesthetized with isoflurane and placed in a standard Kopf stereotactic frame (David Kopf Instruments). Research-grade NSC suspensions (NSI-HK532-IGF-1) were provided by Neuralstem, Inc. at 30k/µL in proprietary hibernation medium (Neuralstem, Inc., Germantown, MD)^50^. Cell viability was determined by standard Trypan Blue exclusion to ensure transplantation of >90% viable cells^50^. NSCs or vehicle only (sham) were delivered by bilateral injection to the fimbria fornix of the hippocampus at 3 sites (6 × 1 µL injections, each administered over 60 s) at the following coordinates (bregma/lateral/ventral): −0.82/0.75/2.5, −1.46/2.3/2.9, −1.94/2.8/2.9 mm, totaling 180k NSC per animal^23^. Sham and NSC groups received the same immunosuppression regimen consisting of subcutaneous mycophenolate (30 mg/kg daily) for 7 d post-grafting and tacrolimus (FK506; 3 mg/kg daily) for the study duration as previously reported^24,49^.

### Behavior testing

Hippocampal-dependent cognitive function was assessed by standard NOR and MWM tasks^51–53^. All animals were hand-habituated for 7 consecutive days before behavioral assessment, and testing was performed by an experienced investigator blinded to genotype and treatment group.

The NOR task was performed over 2 days, at baseline (8 weeks of age) and again 4 weeks post-treatment (16 weeks of age), in an open field, circular polyethylene container (38 cm height × 46 cm diameter) as previously described^51,52^. Mice were tracked and recorded by a digital camera mounted above the open field and LimeLight software (ActiMetrics, Wilmette, IL). Day one consisted of three habituation trials (3 × 240 s) in the empty arena (no objects). Day 2 recognition testing was performed 24 h later, comprising an exploration phase and a test phase. During exploration, mice were placed in the arena and allowed to freely explore two identical objects (300 s). After a 30–45 min delay, mice were returned to the arena, containing one of the original “familiar” objects and a second different “novel” object, and allowed to freely explore (300 s). Objects used were LEGOs^®^ of similar size, fixed equidistant from each other within the arena, approximately 10 cm from the perimeter. Familiar or novel assignment and location was counterbalanced within each experiment. The arena and objects were cleaned between trials with 70% ethanol. Exploration times for familiar and novel objects were calculated by a trained observer using the LimeLight system. Exploration was defined as direct touching of the object, sniffing of the object, or climbing on the object.

Spatial reference memory and learning was assessed by standard MWM performed 16 weeks post-transplant (28 weeks of age) as previously described^51,53^. The maze comprised a 1.2 diameter acrylic pool filled with opaque water at 27 ± 2 °C and a 10 cm escape platform submerged 0.5 cm below the surface. High-contrast posters serving as distal cues were placed on the walls surrounding the pool, and mice were tracked using a digital camera mounted above the pool and WaterMaze software (ActiMetrics). Mice were trained to find the hidden platform in 6 trials per day for 5 days. In each trial, mice were released into the water from pseudo-random starting positions facing the pool wall, and allowed to search until reaching the platform or until 60 s had lapsed. All trials began and ended with 15 s on the platform. To assess memory retention, the platform was removed and a probe trial was conducted on day 6. Each mouse was released into the pool directly opposite the location of the training platform and allowed to swim for 60 s. Following the probe trial, a visible platform test was performed to control for visual acuity, where the escape platform was marked with a distinct local cue. Standard performance measures included latency, time in the TQ and AL, AR, and OP quadrants, and swim speed.

### Tissue analyses

At study end (17 weeks post-transplant), animals were euthanized and perfused with saline. Brains were dissected; one half was snap-frozen for ELISA and one half was fixed in 4% paraformaldehyde and sectioned for IHC (coronal, 40 µm) as described^24^. Briefly, sections were blocked with 10% normal donkey serum, 5% BSA, and 0.3% Triton X-100 in PBS and incubated with primary antibodies (Supplementary Table S1). Antibody targets were visualized with fluorescently-conjugated secondary antibodies (Thermo Fisher Scientific, Hampton, NH).

For IHC quantification, fluorescent images were captured in a blinded manner from dentate, hippocampus, and cortex (5 sections/animal; totaling 25 images/animal) using identical camera settings on a Nikon FX-A microscope (Nikon Instruments, Chiyoda, Japan). Pixel histograms were generated for each image by a custom algorithm (MathWorks, Natick, MA) and used to calculate pixel number and intensity, which was summed for each image providing a composite measure of fluorescent intensity and area for each target. For analysis of activated microglia, IBA-1+ cells that displayed an activated morphology were counted in the same images using ImageJ software (National Institutes of Health). Activated morphology was characterized by a large amoeboid cell body. Additional high-quality representative images were captured on a Leica SP2 confocal (Leica Microsystems, Buffalo Grove, IL).

Human Aβ40/42 and mouse IL-1β and TNF-α were quantified in tissue homogenates by ELISA (Thermo Fisher Scientific and RayBiotech Inc., Norcross, GA) according to manufacturer guidelines.

### *In vitro* microglia-NSC co-culture assay

For *in vitro* experiments, human primary microglia (Applied Biological Materials Inc., Richmond, BC) were cultured according to manufacturer guidelines with and without NSCs (supplied by Neuralstem, Inc.)^23^ in 0.4 µm transwell inserts. Proliferation and phagocytosis were assessed using the ClickIT EdU Imaging Kit (Thermo Fisher Scientific) and uptake of HiLyte™ Fluor 488-labeled human Aβ (1–42) peptide (10 µM; 72 h; AnaSpec, Fremont, CA), respectively. Microglia were stimulated with LPS (50 ng/mL; 2 h) as a positive control, and inserts containing 50B11 cells provided a co-culture control. Fluorescent images were captured (5 images/well; totaling 15–30 images/treatment group) using identical microscope settings on a Nikon Microphot-FXA. EdU+ cells and total nuclei were counted to calculate percent Edu+ proliferating cells. To quantify phagocytosis, a composite measure of fluorescent intensity was calculated by generating pixel histograms as described above.

### Statistical analysis

Statistical analyses were performed using GraphPad Prism 7 (GraphPad Software, Inc., La Jolla, CA). Data are presented as mean ± standard error of the mean (SEM), and significance was determined using an alpha-level of 0.05. Brown-Forsythe F-tests were used to compare variances. Normally distributed data were analyzed by parametric t-test or one-way ANOVA with Tukey’s post-test. Non-normally distributed data were log2 transformed and re-analyzed with the F-test. Non-parametric tests were used on the original data if transformation did not normalize distribution (Mann-Whitney for t-test or Kruskal-Wallis and Dunn’s post-test for multiple comparisons).

Recognition memory in NOR was analyzed using a two-tailed t-test between novel vs. familiar for each group (WT, sham, and NSC). Learning in the MWM was analyzed using repeated measures ANOVA with training (days 1–5) and treatment group (WT, sham and NSC) as factors, and Tukey’s multiple comparisons test. In the probe trial, differences in percentage of time spent in the quadrants (TQ, AL, OP, and AR) were analyzed using one-way ANOVA with Tukey’s multiple comparisons test for each group (WT, sham, and NSC). Platform preference in the probe trial was analyzed using a one-sample t-test, with chance set as the hypothetical mean (25%). Probe trial latency and swim speed were analyzed by one-way ANOVA with Tukey’s multiple comparisons test. Differences between sham and NSC groups in IHC (Aβ, IBA-1, synaptophysin, PSD-95 and ChAT) and ELISA (human Aβ40/42 and mouse IL-1β and TNF-α) were analyzed using an unpaired t-test or Mann-Whitney test for non-normally distributed data. Microglial proliferation and phagocytosis was analyzed using one-way ANOVA with Tukey’s multiple comparisons test for each group (unstimulated, LPS, NSC, 50B11).

## Electronic supplementary material

Supplementary Information

## References

[1] Karlawish J, Jack CR, Rocca WA, Snyder HM, Carrillo MC (2017). Alzheimer’s disease: The next frontier-Special Report 2017. Alzheimer’s & dementia: the journal of the Alzheimer’s Association.

[2] Winblad B (2016). Defeating Alzheimer’s disease and other dementias: a priority for European science and society. The Lancet. Neurology.

[3] Cummings J (2016). Drug development in Alzheimer’s disease: the path to 2025. Alzheimer’s research & therapy.

[4] Cummings JL, Morstorf T, Zhong K (2014). Alzheimer’s disease drug-development pipeline: few candidates, frequent failures. Alzheimer’s research & therapy.

[5] Kalladka D (2016). Human neural stem cells in patients with chronic ischaemic stroke (PISCES): a phase 1, first-in-man study. Lancet.

[6] Steinberg GK (2016). Clinical Outcomes of Transplanted Modified Bone Marrow-Derived Mesenchymal Stem Cells in Stroke: A Phase 1/2a Study. Stroke; a journal of cerebral circulation.

[7] Garitaonandia I (2016). Neural Stem Cell Tumorigenicity and Biodistribution Assessment for Phase I Clinical Trial in Parkinson’s Disease. Sci Rep.

[8] Chen KS, Sakowski SA, Feldman EL (2016). Intraspinal stem cell transplantation for amyotrophic lateral sclerosis. Annals of neurology.

[9] Feldman EL (2014). Intraspinal neural stem cell transplantation in amyotrophic lateral sclerosis: Phase 1 trial outcomes. Ann Neurol.

[10] Glass JD (2016). Transplantation of spinal cord-derived neural stem cells for ALS: Analysis of phase 1 and 2 trials. Neurology.

[11] Riley J (2014). Intraspinal stem cell transplantation in amyotrophic lateral sclerosis: a phase I trial, cervical microinjection, and final surgical safety outcomes. Neurosurgery.

[12] Tadesse T (2014). Analysis of graft survival in a trial of stem cell transplant in ALS. Annals of clinical and translational neurology.

[13] Goutman, S. A. *et al*. Long-term Phase 1/2 intraspinal stem cell transplantation outcomes in amyotrophic lateral sclerosis. *Annals of clinical and translational neurology* In Press (2018). 10.1002/acn3.567 PMC5989736 29928656

[14] Ager Rahasson R., Davis Joy L., Agazaryan Andy, Benavente Francisca, Poon Wayne W., LaFerla Frank M., Blurton-Jones Mathew (2015). Human neural stem cells improve cognition and promote synaptic growth in two complementary transgenic models of Alzheimer's disease and neuronal loss. Hippocampus.

[15] Blurton-Jones M (2009). Neural stem cells improve cognition via BDNF in a transgenic model of Alzheimer disease. Proceedings of the National Academy of Sciences of the United States of America.

[16] Lee HJ (2012). Human umbilical cord blood-derived mesenchymal stem cells improve neuropathology and cognitive impairment in an Alzheimer’s disease mouse model through modulation of neuroinflammation. Neurobiology of aging.

[17] Lee IS (2015). Human neural stem cells alleviate Alzheimer-like pathology in a mouse model. Molecular neurodegeneration.

[18] Lee JK, Jin HK, Bae JS (2009). Bone marrow-derived mesenchymal stem cells reduce brain amyloid-beta deposition and accelerate the activation of microglia in an acutely induced Alzheimer’s disease mouse model. Neuroscience letters.

[19] Lee JK (2010). Intracerebral transplantation of bone marrow-derived mesenchymal stem cells reduces amyloid-beta deposition and rescues memory deficits in Alzheimer’s disease mice by modulation of immune responses. Stem cells.

[20] Garcia KO (2014). Therapeutic effects of the transplantation of VEGF overexpressing bone marrow mesenchymal stem cells in the hippocampus of murine model of Alzheimer’s disease. Frontiers in aging neuroscience.

[21] Duncan T, Valenzuela M (2017). Alzheimer’s disease, dementia, and stem cell therapy. Stem cell research & therapy.

[22] Kim HJ (2015). Stereotactic brain injection of human umbilical cord blood mesenchymal stem cells in patients with Alzheimer’s disease dementia: A phase 1 clinical trial. Alzheimers Dement (Amst).

[23] McGinley LM (2016). Human Cortical Neural Stem Cells Expressing Insulin-Like Growth Factor-I: A Novel Cellular Therapy for Alzheimer’s Disease. Stem cells translational medicine.

[24] McGinley LM (2017). Human neural stem cell transplantation into the corpus callosum of Alzheimer’s mice. Annals of clinical and translational neurology.

[25] Johe KK, Hazel TG, Muller T, Dugich-Djordjevic MM, McKay RD (1996). Single factors direct the differentiation of stem cells from the fetal and adult central nervous system. Genes Dev.

[26] Yang H (2013). Human umbilical cord mesenchymal stem cell-derived neuron-like cells rescue memory deficits and reduce amyloid-beta deposition in an AbetaPP/PS1 transgenic mouse model. Stem cell research & therapy.

[27] Hunsberger JG (2016). Accelerating stem cell trials for Alzheimer’s disease. The Lancet. Neurology.

[28] Kim KS (2013). Long-term immunomodulatory effect of amniotic stem cells in an Alzheimer’s disease model. Neurobiology of aging.

[29] Naaldijk Y (2017). Effect of systemic transplantation of bone marrow-derived mesenchymal stem cells on neuropathology markers in APP/PS1 Alzheimer mice. Neuropathol Appl Neurobiol.

[30] Oh SH, Kim HN, Park HJ, Shin JY, Lee PH (2015). Mesenchymal Stem Cells Increase Hippocampal Neurogenesis and Neuronal Differentiation by Enhancing the Wnt Signaling Pathway in an Alzheimer’s Disease Model. Cell transplantation.

[31] Yun HM (2013). Placenta-derived mesenchymal stem cells improve memory dysfunction in an Abeta1-42-infused mouse model of Alzheimer’s disease. Cell Death Dis.

[32] Fujiwara Naruyoshi, Shimizu Jun, Takai Kenji, Arimitsu Nagisa, Saito Asako, Kono Takao, Umehara Tasuku, Ueda Yuji, Wakisaka Sueshige, Suzuki Tomoko, Suzuki Noboru (2013). Restoration of spatial memory dysfunction of human APP transgenic mice by transplantation of neuronal precursors derived from human iPS cells. Neuroscience Letters.

[33] Cramer PE (2012). ApoE-directed therapeutics rapidly clear beta-amyloid and reverse deficits in AD mouse models. Science.

[34] Yang H, Yang H, Xie Z, Wei L, Bi J (2013). Systemic transplantation of human umbilical cord derived mesenchymal stem cells-educated T regulatory cells improved the impaired cognition in AbetaPPswe/PS1dE9 transgenic mice. PloS one.

[35] Guo W, Sha S, Xing X, Jiang T, Cao Y (2013). Reduction of cerebral Abeta burden and improvement in cognitive function in Tg-APPswe/PSEN1dE9 mice following vaccination with a multivalent Abeta3-10 DNA vaccine. Neuroscience letters.

[36] Gu G, Zhang W, Li M, Ni J, Wang P (2015). Transplantation of NSC-derived cholinergic neuron-like cells improves cognitive function in APP/PS1 transgenic mice. Neuroscience.

[37] Zhang Wei, Wang Pei-Jun, Sha Hong-ying, Ni Jiong, Li Ming-hua, Gu Guo-jun (2014). Neural Stem Cell Transplants Improve Cognitive Function Without Altering Amyloid Pathology in an APP/PS1 Double Transgenic Model of Alzheimer’s Disease. Molecular Neurobiology.

[38] El Khoury J, Hickman SE, Thomas CA, Loike JD, Silverstein SC (1998). Microglia, scavenger receptors, and the pathogenesis of Alzheimer’s disease. Neurobiology of aging.

[39] Leissring MA (2003). Enhanced proteolysis of beta-amyloid in APP transgenic mice prevents plaque formation, secondary pathology, and premature death. Neuron.

[40] Yan P (2006). Matrix metalloproteinase-9 degrades amyloid-beta fibrils *in vitro* and compact plaques *in situ*. J Biol Chem.

[41] Zhao Z (2007). Insulin degrading enzyme activity selectively decreases in the hippocampal formation of cases at high risk to develop Alzheimer’s disease. Neurobiology of aging.

[42] Zanier ER (2014). Bone marrow mesenchymal stromal cells drive protective M2 microglia polarization after brain trauma. Neurotherapeutics.

[43] Noh MY (2016). Mesenchymal Stem Cells Modulate the Functional Properties of Microglia via TGF-beta Secretion. Stem cells translational medicine.

[44] El Khoury J (2007). Ccr2 deficiency impairs microglial accumulation and accelerates progression of Alzheimer-like disease. Nature medicine.

[45] Richard KL, Filali M, Prefontaine P, Rivest S (2008). Toll-like receptor 2 acts as a natural innate immune receptor to clear amyloid beta 1-42 and delay the cognitive decline in a mouse model of Alzheimer’s disease. The Journal of neuroscience: the official journal of the Society for Neuroscience.

[46] Lee JK, Schuchman EH, Jin HK, Bae JS (2012). Soluble CCL5 derived from bone marrow-derived mesenchymal stem cells and activated by amyloid beta ameliorates Alzheimer’s disease in mice by recruiting bone marrow-induced microglia immune responses. Stem cells.

[47] Hefferan MP (2012). Human neural stem cell replacement therapy for amyotrophic lateral sclerosis by spinal transplantation. PloS one.

[48] Cheng K, Gupta S (2009). Quantitative tools for assessing the fate of xenotransplanted human stem/progenitor cells in chimeric mice. Xenotransplantation.

[49] Hefferan MP (2011). Optimization of immunosuppressive therapy for spinal grafting of human spinal stem cells in a rat model of ALS. Cell transplantation.

[50] Glass JD (2012). Lumbar intraspinal injection of neural stem cells in patients with amyotrophic lateral sclerosis: results of a phase I trial in 12 patients. Stem cells.

[51] Sims-Robinson C (2016). Dietary Reversal Ameliorates Short- and Long-Term Memory Deficits Induced by High-fat Diet Early in Life. PloS one.

[52] Moore SJ, Deshpande K, Stinnett GS, Seasholtz AF, Murphy GG (2013). Conversion of short-term to long-term memory in the novel object recognition paradigm. Neurobiology of learning and memory.

[53] Temme, S. J., Bell, R. Z., Fisher, G. L. & Murphy, G. G. Deletion of the Mouse Homolog of CACNA1C Disrupts Discrete Forms of Hippocampal-Dependent Memory and Neurogenesis within the Dentate Gyrus. *eNeuro* **3**, 10.1523/ENEURO.0118-16.2016 (2016). 10.1523/ENEURO.0118-16.2016 PMC5124786 27957527

