# Supplementary material for: Human neural stem cell transplantation improves cognition in a murine model of Alzheimer’s disease
Source: Sci Rep. 2018 Oct 3;8:14776. doi: 10.1038/s41598-018-33017-6 (PMC6170460; doi:10.1038/s41598-018-33017-6)
Supplement: Supplementary file 1 — Supplementary Information [file 41598_2018_33017_MOESM1_ESM.pdf]

Supplementary Information

Human neural stem cell transplantation improves cognition in a murine model of Alzheimer's disease

Authors: Lisa M. McGinley Ph.D.[1], Osama N. Kashlan M.D.[2], Elizabeth S. Bruno B.S.[1], Kevin S. Chen M.D.[2], John M. Hayes B.S.[1], Samy R. Kashlan M.D.[1], Julia Raykin Ph.D.[3], Karl Johe Ph.D.[4], Geoffrey G. Murphy Ph.D.[5] and Eva L. Feldman M.D. Ph.D.*[1]

Affiliations:

[1] Department of Neurology, University of Michigan, Ann Arbor, MI

[2] Department of Neurosurgery, University of Michigan, Ann Arbor, MI

[3] Department of Biomedical Engineering, Georgia Institute of Technology, Atlanta, GA

[4] Neuralstem, Inc., Germantown, MD

[5] Department of Molecular & Integrative Physiology, Molecular & Behavioral Neuroscience Institute, University of Michigan, Ann Arbor, MI

Address correspondence to:

Eva L. Feldman, M.D. Ph.D., 5017 AAT-BSRB, 109 Zina Pitcher Place, Ann Arbor, MI 48109

Phone: 734-763-7274 / Fax: 734-763-7275

Email: efeldman@umich.edu

Table S1: List of primary antibodies used for immunohistochemistry (IHC).

Antibody	Vendor	Cat. #	Species	Dilution
**Aβ**	Abcam	Ab10148	Rabbit	1:1500
**ChAT**	Millipore	AB-144-P	Rabbit	1:100
**IBA-1**	Wako	019-19741	Rabbit	1:500
**PSD95**	Abcam	Ab18258	Rabbit	1:100
**Synaptophysin**	Abcam	Ab32127	Rabbit	1:500

Supplementary Figure S1: Effect of NSC transplantation on synapse density, cholinergic neurons, and pro-inflammatory cytokines. Representative IHC images (a) and quantification of IHC fluorescent intensity of synaptophysin (b), PSD-95 (c), and ChAT (d) in APP/PS1 mice at 29 weeks (17 weeks post-NSC/vehicle transplant). Synapse density and ChAT levels were unchanged by NSC transplantation, with no significant differences detected between groups (synaptophysin; $p = 0.1297$; PSD-95; $p = 0.4580$; ChAT; $p = 0.1539$; NSC vs. sham; t-test). ELISA quantification on whole brain homogenate of IL-1β (e) and TNF-α (f) shows equivalent levels between treatment groups (IL-1β; $p = 0.4082$ and TNF-α; $p = 0.2115$; NSC vs. sham, Mann-Whitney test). Data are representative images or mean ± SEM. Scale bar 100 μm. Sample size: WT n=5, NSC n=10, and sham n=10.

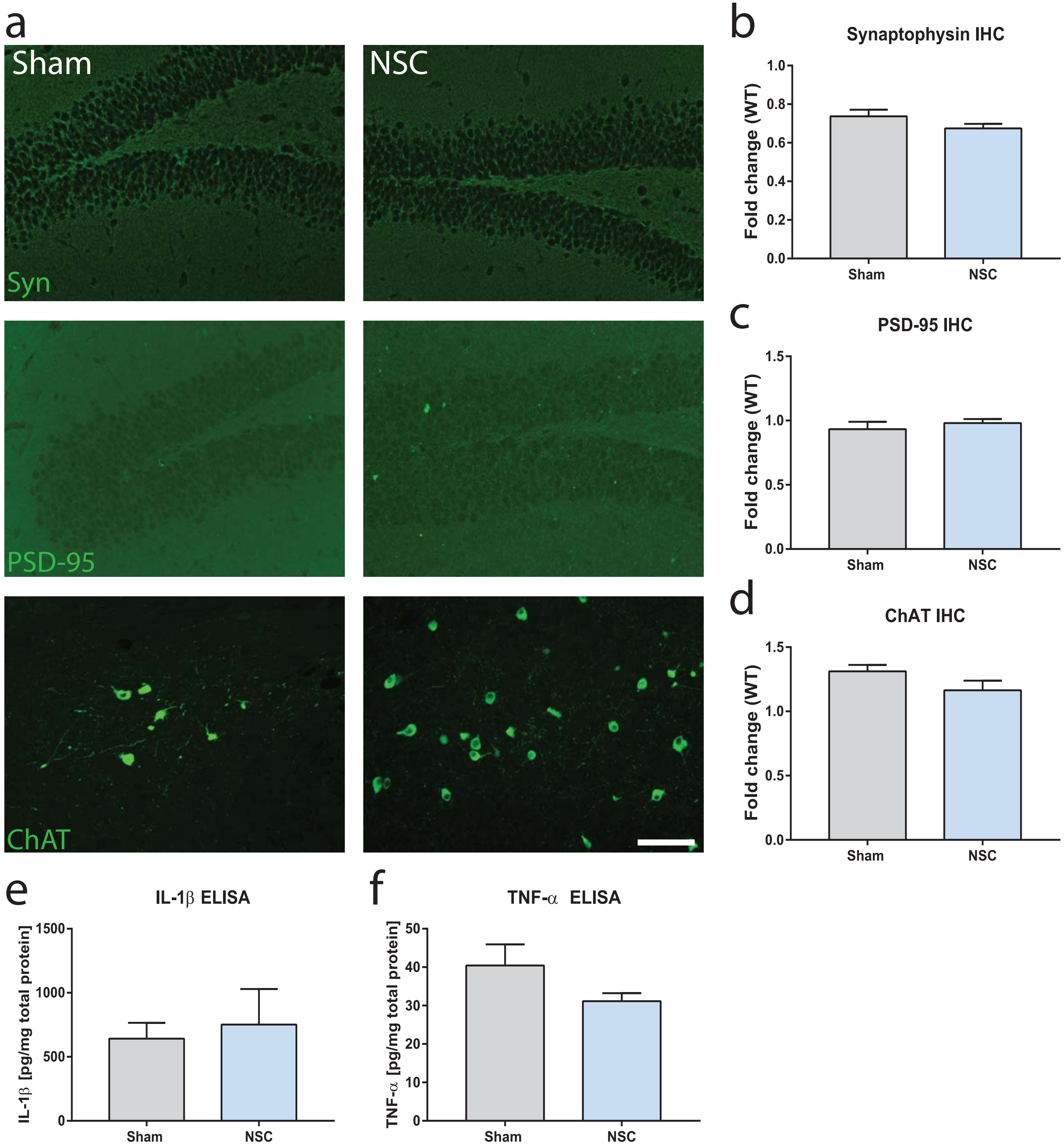
a
Sham
NSC
Syn
PSD-95
ChAT
b
Synaptophysin IHC
Fold change (WT)
1.0
0.8
0.6
0.4
0.2
0.0
Sham
NSC
c
PSD-95 IHC
Fold change (WT)
1.5
1.0
0.5
0.0
Sham
NSC
d
ChAT IHC
Fold change (WT)
1.5
1.0
0.5
0.0
Sham
NSC
e
IL-1β ELISA
IL-1β [pg/mg total protein]
1500
1000
500
0
Sham
NSC
f
TNF-α ELISA
TNF-α [pg/mg total protein]
50
40
30
20
10
0
Sham
NSC
